# Effects of the Btk-Inhibitors Remibrutinib (LOU064) and Rilzabrutinib (PRN1008) With Varying Btk Selectivity Over Tec on Platelet Aggregation and *in vitro* Bleeding Time

**DOI:** 10.3389/fcvm.2021.749022

**Published:** 2021-09-24

**Authors:** Rundan Duan, Luise Goldmann, Richard Brandl, Michael Spannagl, Christian Weber, Wolfgang Siess, Philipp von Hundelshausen

**Affiliations:** ^1^Institute for Cardiovascular Prevention, Ludwig-Maximilians-University, Munich, Germany; ^2^Institute for Vascular Surgery and Phlebology am Marienplatz, Munich, Germany; ^3^Department of Transfusion Medicine, Cell Therapeutics and Hemostaseology, Ludwig-Maximilians University, Munich, Germany; ^4^German Centre for Cardiovascular Research, Deutsches Zentrum für Herz-Kreislauf-Forschung (DZHK), Partner Site Munich Heart Alliance, Munich, Germany; ^5^Department of Biochemistry, Cardiovascular Research Institute Maastricht, Maastricht University, Maastricht, Netherlands

**Keywords:** atherothrombosis, platelet-aggregation, bleeding, PFA, MEA

## Abstract

**Background:** Bruton tyrosine kinase inhibitors (BTKi) are used in B-cell malignancies and in development against various autoimmune diseases. Since Btk is also involved in specific pathways of platelet activation, BTKi might be considered to target platelet GPVI/GPIb-mediated atherothrombosis and platelet FcγRIIA-dependent immune disorders. However, BTKi treatment of patients with B-cell malignancies is frequently associated with mild bleeding events caused possibly by off-target inhibition of Tec. Here, we compared the platelet effects of two novel BTKi that exhibit a high (remibrutinib) or low (rilzabrutinib) selectivity for Btk over Tec.

**Methods and Results:** Remibrutinib and rilzabrutinib were pre-incubated with anticoagulated blood. Platelet aggregation and *in vitro* bleeding time (closure time) were studied by multiple electrode aggregometry (MEA) and platelet-function analyzer-200 (PFA-200), respectively. Both BTKi inhibited atherosclerotic plaque-stimulated GPVI-mediated platelet aggregation, remibrutinib being more potent (IC_50_ = 0.03 μM) than rilzabrutinib (IC_50_ = 0.16 μM). Concentrations of remibrutinib (0.1 μM) and rilzabrutinib (0.5 μM), >80% inhibitory for plaque-induced aggregation, also significantly suppressed (>90%) the Btk-dependent pathways of platelet aggregation upon GPVI, von Willebrand factor/GPIb and FcγRIIA activation stimulated by low collagen concentrations, ristocetin and antibody cross-linking, respectively. Both BTKi did not inhibit aggregation stimulated by ADP, TRAP-6 or arachidonic acid. Remibrutinib (0.1 μM) only slightly prolonged closure time and significantly less than rilzabrutinib (0.5 μM).

**Conclusion:** Remibrutinib and rilzabrutinib inhibit Btk-dependent pathways of platelet aggregation upon GPVI, VWF/GPIb, and FcγRIIA activation. Remibrutinib being more potent and showing a better profile of inhibition of Btk-dependent platelet activation vs. hemostatic impairment than rilzabrutinib may be considered for further development as an antiplatelet drug.

## Introduction

Since the first description of a patient with recurrent infections and deficiency of immunoglobulins termed “Agammaglobulinemia” by Ogden Bruton in 1952 (1), it took more than 40 years of research, until Bruton tyrosine kinase (Btk) was identified in 1993 as the responsible protein that is deficient in patients with X-linked agammaglobulinemia (2, 3). Btk belongs to the Tec (tyrosine kinase expressed in hepatocellular carcinoma) family of non-receptor cytoplasmic tyrosine kinase, and contains five different protein interaction domains: an amino terminal pleckstrin homology (PH) domain, a proline-rich Tec homology (TH) domain, the SRC kinase homology (SH) domains SH2 and SH3, and a kinase domain (4). Btk is the best studied member of this tyrosine kinase family and most homologous to Tec, the namesake of this kinase family. Btk plays a crucial role in B-cell receptor function and in immunoglobulin Fc- receptor signaling of monocytes/macrophages and neutrophils (4). Since the approval of ibrutinib, the covalent irreversible first in class Btk inhibitor (BTKi) in 2013 for treatment of certain B-cell malignancies, many more reversible and irreversible BTKi have evolved and the spectrum of diseases that are targeted extends from specific forms of B-cell malignancies to various autoimmune disorders (5). Up to now four BTKi (ibrutinib, acalabrutinib, zanubrutinib, and tirabrutinib) have been approved but at least further eight BTKi are in clinical studies (5).

Btk is expressed not only in B-cells but also in various hematopoietic cells including the megakaryocyte-platelet lineage (6). Btk in platelets is involved in signaling of specific glycoprotein receptors including glycoprotein (GP) VI activation by collagen, GPIb activation by von Willebrand factor (VWF), FcγRIIa activation by IgG immune complexes, and CLEC-2 activation by podoplanin (5). Thus, Btk might be a promising therapeutic target of platelet-related diseases, and BTKi have indeed been proposed as novel antiplatelet drugs as they inhibit selectively platelet GPVI/GPIb-stimulated atherothrombosis (7, 8), platelet FcγRIIA-dependent immune disorders (heparin-induced thrombocytopenia, vaccine-induced immune thrombotic thrombocytopenia) (9, 10), and podoplanin/CLEC-2 mediated venous thrombosis (11, 12). Somewhat surprisingly, XLA patients do not show a bleeding tendency (13). In contrast, mild bleeding events are frequent in patients with B-cell malignancies treated with irreversible covalent BTKi (ibrutinib, acalabrutinib, zanubrutinib, and tirabrutinib) (5). The reasons are not clear but are probably multifactorial. They might be related to the type of diseases treated, but also caused by off-target inhibition as reviewed recently (5).

Beside Btk the homologous kinase Tec is also expressed in platelets. Whereas, FcγRIIa activation and VWF activation of GPIb do not require Tec activation (5, 9), Tec plays a role in GPVI activation. After GPVI-mediated platelet stimulation by high dose collagen, both Btk and Tec support platelet aggregation. Btk-deficient human platelets from patients with XLA and Btk-deficient mouse platelets do not respond to low concentrations of collagen or collagen-related peptide (CRP) indicating that Btk is required for platelet activation after low-degree GPVI stimulation (14, 15). Similar observations have been made by using low Btk-specific concentrations of irreversible BTKi and the reversible BTKi fenebrutinib which inhibits Btk but not Tec and applying human atherosclerotic plaque which also induces only a low-degree activation of GPVI (8, 9, 16–18). After stimulation with high concentrations of collagen, Tec compensates for the absence of Btk (as in XLA patients) or inhibition of Btk (as after platelet pretreatment with Btk-selective concentrations of BTKi) and preserves GPVI-stimulated platelet aggregation. Inhibition of both Tec and Btk abrogates GPVI-activation (15). Since the four approved irreversible covalent BTKi mentioned above have limited selectivity for Btk over Tec as measured by biochemical assays *in vitro* (5), and at higher concentrations prolong bleeding time *in vitro* (19), it is assumed that therapeutic concentrations of these BTKi inhibit in platelets irreversibly Tec in addition to Btk thereby abrogating GPVI signaling. This might contribute to the observed bleeding side effects.

Therefore, we hypothesized that off target effects of BTKi with low Btk selectivity over Tec might explain bleeding of BTKi, and investigated in the present study the effects of two novel BTKi on Btk-mediated pathways of platelet aggregation and bleeding time *in vitro*: the novel selective covalent BTKi remibrutinib (LOU064), a very potent irreversible covalent BTKi, which is highly selective for Btk and barely inhibits Tec (20), and rilzabrutinib (PRN1008) an oral, reversible covalent BTKi which inhibits Btk and Tec with similar IC50 values (21). Both BTKi are in clinical studies of dermatological autoimmune diseases. Rilzabrutinib inhibits very potently Btk and Tec *in vitro* (IC50 values, 1.3 and 0.8 nM, respectively) (22). It forms a reversible covalent bond with Cys481 of Btk, and shows a fast association and a very slow dissociation rate (23). Rilzabrutinib is in clinical trials of pemphigus (24) and idiopathic thrombocytopenic purpura (ITP), a disease exhibiting very low platelet counts (<50.000/μl) and bleeding events. Here, it inhibits platelet destruction mainly via the inhibition of autoantibody/FcγR signaling in splenic macrophages (25). Unexpectedly, in a previous report clinically relevant concentrations of rilzabrutinib showed no inhibition of platelet activation *in vitro* (26).

## Materials and Methods

### Reagents

Remibrutinib (LOU064), rilzabrutinib (PRN1008) and fenebrutinib (GDC-0853) were purchased from MedChem Express (New Jersey, USA). Dimethyl sulfoxide (DMSO) was from Sigma-Aldrich (Taufkirchen, Germany). Collagen was from Takeda (Linz, Austria). ADP, ristocetin, arachidonic acid (AA) and TRAP-6 (Thrombin Receptor Activator Peptide 6) were obtained from Roche Diagnostics (Mannheim, Germany). The anti-CD32 antibody AT10 (monoclonal mouse IgG1), cross-adsorbed F(ab')2-goat anti-mouse IgG (H + L) and the anti-CD9 antibody Ts9 (monoclonal mouse IgG1) were from ThermoFisher Scientific (Waltham, MA, USA).

### Declaration of Helsinki

Informed consent was obtained from healthy volunteers, as approved by the Ethics Committee of the Faculty of Medicine of the University of Munich, and in accordance with the ethical principles for medical research involving human subjects, as set out in the Declaration of Helsinki.

### Human Atherosclerotic Plaque Homogenates

Atherosclerotic tissue specimens were obtained from patients who underwent endarterectomy for high-grade carotid artery stenosis. Specimen containing lipid-rich soft plaques were collected. The atheromatous plaques were carefully dissected under sterile conditions from other regions of the atherosclerotic tissue. The plaques were weighed, homogenized with a glass pestle and potter, then stored at −80°C (27, 28). Plaque homogenates from 5 patients were pooled.

### Blood Collection

Whole blood from healthy donors who had not taken any antiplatelet drug within 2 weeks was collected by cubital venipuncture into blood tubes (double wall) from Verum Diagnostica GmbH (Munich, Germany) containing hirudin as anticoagulant (final hirudin concentration in blood: 200 U/ml corresponding to 15 μg/ml) for platelet aggregation measurements (29) or buffered trisodium citrate/citric acid solution (citrate concentration 0.129 mol/L; S-Monovette 3.8 mL 9NC/PFA from Sarstedt, Nümbrecht, Germany) for closure time measurements with the PFA-200 (30). The blood was kept at room temperature and measurements were performed with 3 h after venipuncture.

### Platelet Aggregation in Blood

Multiple electrode aggregometry (MEA) (Roche Diagnostics, Mannheim, Germany) that monitors the change of conductivity between two sets of electrodes (red and blue traces) caused by the attachment of platelets was applied to measure platelet aggregation, as described (29, 31). In brief, 0.9% NaCl (300 μL) was placed in aggregometer cuvettes (06675590, Roche, Mannheim, Germany) with aliquots (300 μL) of hirudin-anticoagulated blood. BTKi or DMSO (solvent control; 0.6 μL) were added, and mixed well with pipet, covered, and incubated for 1 h at 37°C (19). Then, the cuvettes were transferred into the device, platelet stimuli (collagen, ristocetin, AT10 + Fab2, anti-CD9 antibody, TRAP-6, ADP, or AA) were added at concentrations as detailed in the figure legends, stirring was simultaneously started and aggregation was measured for 10 min. Aggregation was recorded in arbitrary units (AU), and cumulative aggregation (AU^*^min) from 0 to 10 min was measured by quantifying the area under the curve. The traces selected as representative and displayed in the Figures belonged to a specific experiment whose values were closest to the mean.

IC50 values were obtained by non-linear fitting using the following model:

Fifty = (Top + Baseline)/2
Y = Bottom + (Top-Bottom)/(1 + 10^∧^((LogAbsoluteIC50-X)*HillSlope + log((Top-Bottom)/(Fifty-Bottom)-1)))

### Closure Time Measurement

The INNOVANCE^®^ PFA-200 System (Siemens Healthcare, Erlangen, Germany), which simulates primary hemostasis *in vitro*, provides rapid and precise assessment of platelet dysfunction and bleeding risk (32, 33). DMSO (0.1%; solvent control) or various concentrations of remibrutinib or rilzabrutinib were pipetted (0.8 μl) into samples of citrate-anticoagulated blood (0.8 ml) (30) and preincubated for 1 h at 37°C. The Dade^®^ PFA Collagen/EPI Test Cartridge was used, and the time of complete plug formation was reported as “closure time.” The normal range of closure time is assessed specifically for each test center and was determined to be 84–170 s. The normal range as recommended by the manufacturer (84–160 s) has been slightly modified at our institution to 84–170 s based on the measurement on 54 healthy unselected persons without any medication according to the approved-level consensus guideline from the Clinical and Laboratory Standards Institute (CLSI EP28).

### Statistics

The data are shown as mean ± standard deviation (SD) of the indicated number of the experiments. Normal distribution of values was assessed using the Shapiro-Wilk test. Parallel multi-experimental conditions were analyzed by ordinary one-way ANOVA, followed by Bonferroni's test if the normality test was passed, otherwise a Kruskal-Wallis test for unmatched and a Friedman's test for matched observations followed by Dunn's test were used. Side-by side comparisons were analyzed by Wilcoxon matched-pairs signed rank test.

## Results

### Remibrutinib (LOU064) and Rilzabrutinib (PRN1008) Dose-Dependently Inhibit GPVI-Mediated Platelet Aggregation in Blood Triggered by Atherosclerotic Plaque

**Figure 1:**
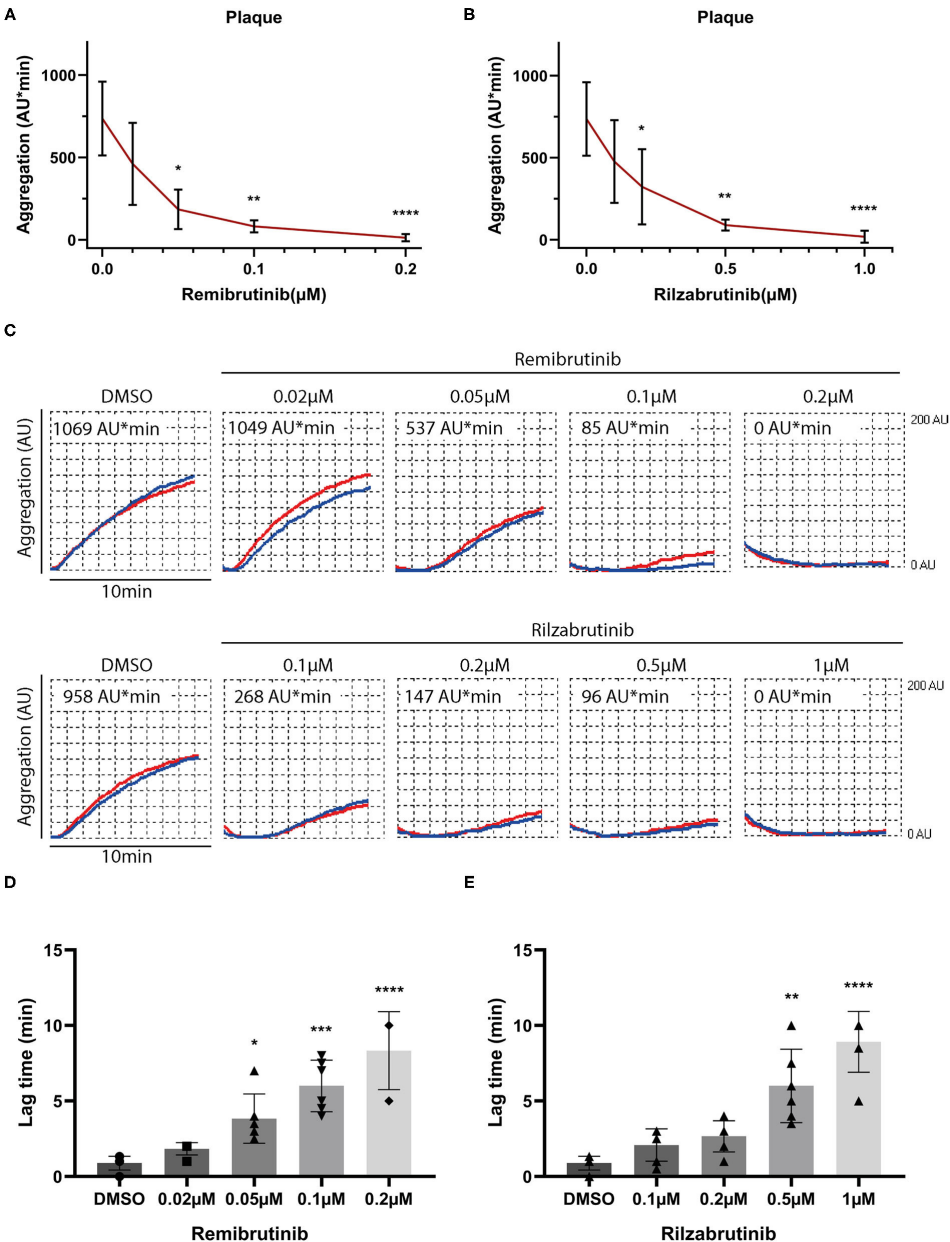
Effects of increasing concentrations of remibrutinib and rilzabrutinib on platelet aggregation in blood stimulated by plaque homogenate. Hirudin anticoagulated blood was preincubated for 1 h at 37°C with solvent control (DMSO, 0.1%), or increasing concentrations of remibrutinib **(A,C,D)** or rilzabrutinib **(B,C,E)** and aggregation was recorded for 10 min after stimulation by plaque homogenate (833 μg/ml) (19). The dose-response curves of **(A)** remibrutinib and **(B)** rilzabrutinib are shown. **(C)** Representative aggregation traces in red and blue for each electrode, respectively, are shown. **(D,E)** Bar graphs show the dose-dependent delay in aggregation by **(D)** remibrutinib and **(E)** rilzabrutinib. Single data points are shown but are in part not visible due to overlap. Values are mean ± SD (*n* = 6). Statistical analysis was carried out comparing against baseline (without BTKi) using the Friedman test followed by Dunn's test **(A–E)**. ^*^p < 0.05, ^**^p < 0.01, ^***^p < 0.001, ^****^p < 0.0001.

Diverse collagen type I and III fibers are the decisive plaque components that induce platelet aggregation via activation of GPVI (27, 28, 34). Blood was incubated with increasing concentrations of remibrutinib or rilzabrutinib for 1 h prior to plaque stimulation. Remibrutinib and rilzabrutinib inhibited plaque-induced platelet aggregation with IC_50_ values of 0.03 and 0.16 μM, respectively. Remibrutinib (0.2 μM) and rilzabrutinib (1 μM) were able to block plaque-induced platelet aggregation by >90% (Figures 1A,B). Accordingly, remibrutinib is more potent than rilzabrutinib.

The aggregation tracings in Figure 1C and panels in Figures 1D,E show a dose-dependent increase in delay of aggregation (lag time) caused by both inhibitors.

### Effects of Remibrutinib and Rilzabrutinib on Platelet Aggregation Stimulated by Collagen, Ristocetin, FcγRIIA- and G-Protein Coupled Receptor-Activation

Next the effects of remibrutinib and rilzabrutinib were investigated on platelet aggregation induced by stimuli known to activate Btk-dependent and Btk-independent platelet signaling pathways. Concentrations of remibrutinib (0.1 μM) and rilzabrutinib (0.5 μM) were chosen that inhibited atherosclerotic plaque-induced platelet aggregation by 89 and 88%, respectively (Figure 1A).

**Figure 2:**
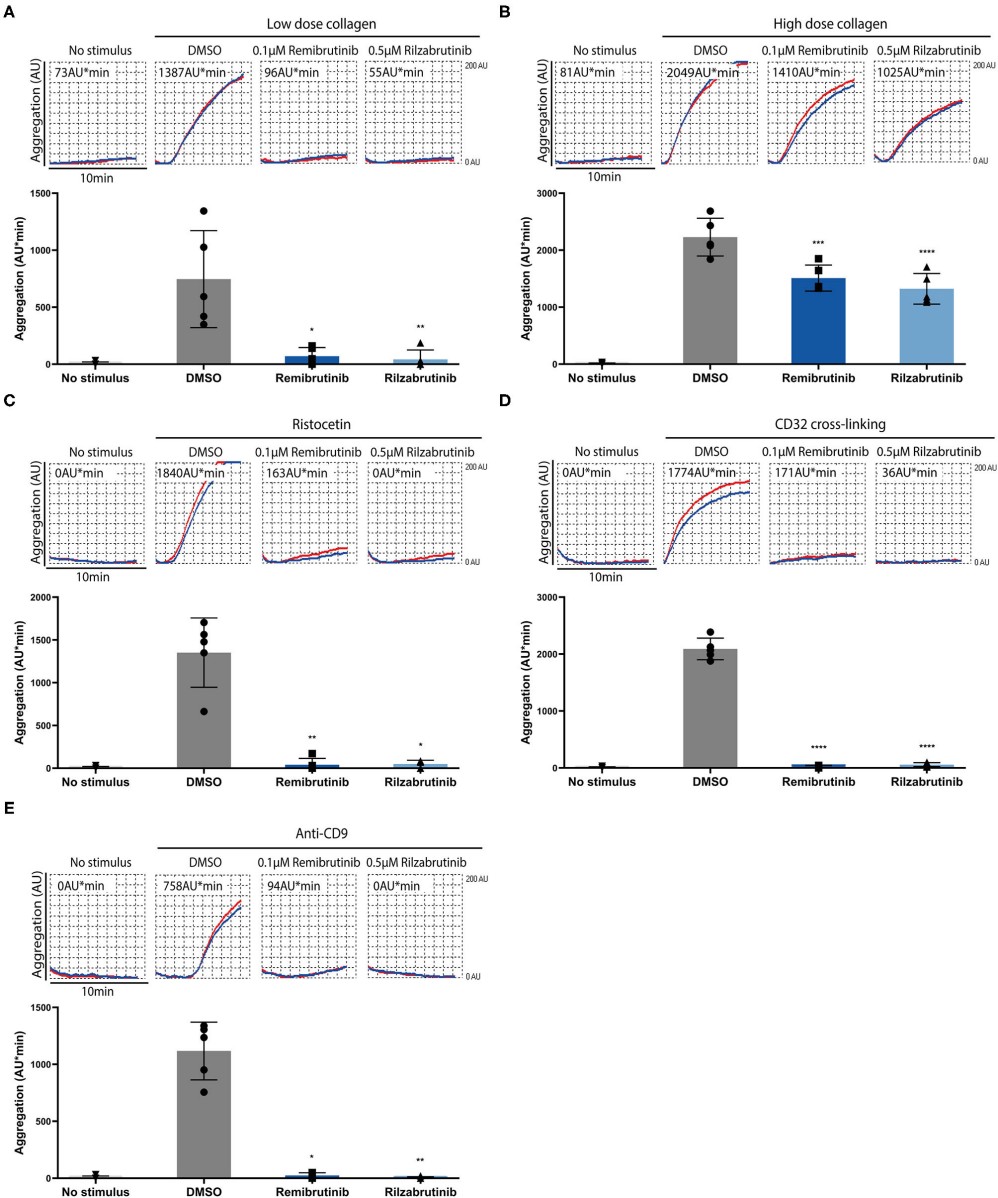
Effects of remibrutinib and rilzabrutinib on platelet aggregation in blood after stimulation by collagen, ristocetin or FcγRIIA activation. Hirudin anticoagulated blood was preincubated for 1 h with DMSO or BTKi (remibrutinib 0.1 μM, rilzabrutinib 0.5 μM) prior to stimulation with **(A)** low dose collagen (0.4–0.6 μg/ml) that was titrated to induce a similar degree of platelet aggregation as plaque homogenate (833 μg/mL) (19), **(B)** high dose collagen (4–6 μg/ml) that was 10× concentrations of the low dose collagen (8, 19), **(C)** ristocetin (0.5 mg/ml), **(D)** CD32 cross-linking (3 min incubation with 2 μg/ml AT10, plus 30 μg/ml Fab2), or **(E)** anti-CD9 antibody (1 μg/ml). Representative MEA tracings (top panels) and bar graphs (bottom panels) are shown. Values are shown as mean ± SD (*n* = 5). Statistical analysis was carried out using ordinary one-way ANOVA followed by Bonferroni's test **(B,D)** or Kruskal-Wallis followed by Dunn's test **(A,C,E)**. ^*^p < 0.05, ^**^p < 0.01, ^***^p < 0.001, ^****^p < 0.0001.

Figure 2 shows the results for platelet stimuli that induce aggregation through a Btk-dependent mechanism (5). GPVI-dependent aggregation was inhibited by remibrutinib and rilzabrutinib by 91 and 94%, respectively, on low dose collagen, and by 37 and 41%, respectively, on high dose collagen (Figures 2A,B). Glycoprotein Ib/von Willebrand factor (GPIb/VWF)-dependent ristocetin-induced platelet aggregation was blocked by 95% by both BTKi (Figure 2C). The inhibitory effects of remibrutinib and rilzabrutinib on GPVI- and GPIb/VWF-dependent platelet aggregation were similar to those of fenebrutinib (0.1 μM) (Supplementary Figure 2), which is a reversible and highly selective Btk inhibitor.

Complete suppression of platelet aggregation by both BTKi was also observed on FcγRIIA activation by crosslinking or anti-CD9 antibody stimulation (Figures 2D,E). Due to the absence of adenosine 5'-diphosphate (ADP) secretion from platelets (9), anti-CD9 antibody stimulation showed a delayed aggregation response and less maximal aggregation compared with CD32-crosslinking (Figure 2E).

**Figure 3:**
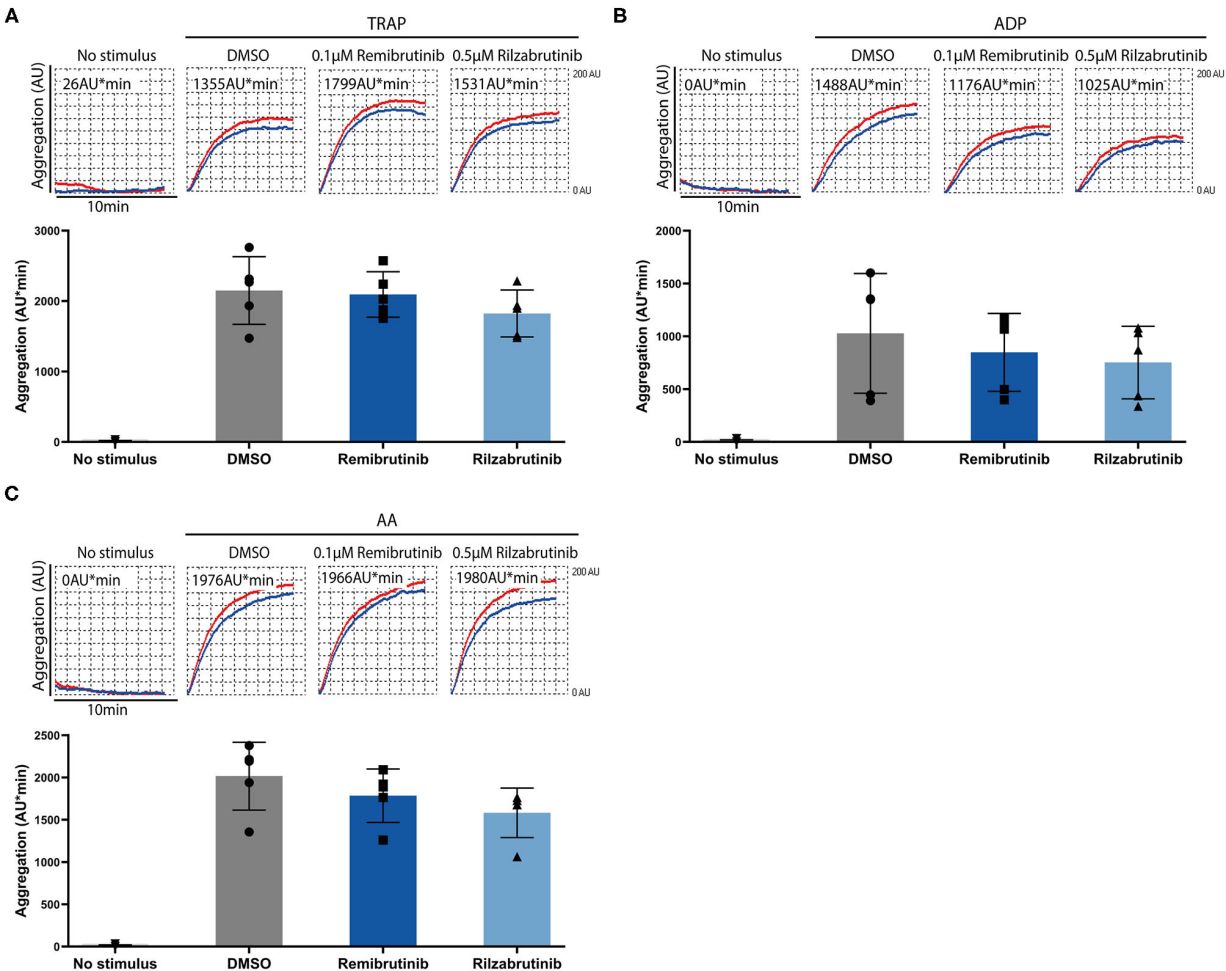
Effects of remibrutinib and rilzabrutinib on platelet aggregation in blood after stimulation by TRAP, ADP, or AA. Hirudin anticoagulated blood samples were pretreated for 1 h at 37°C with solvent control (DMSO, 0.1%), or BTKi (remibrutinib 0.1 μM, rilzabrutinib 0.5 μM) before stimulation with **(A)** TRAP (15 μM), **(B)** ADP (10 μM), or **(C)** AA (0.6 mM). Representative MEA tracings (top panels) and aligned dot blot bar graphs (bottom panels) are shown. Values shown are mean ± SD (*n* = 5). Statistical analysis was carried out using ordinary ANOVA followed by Bonferroni's test **(A,B)** or Kruskal-Wallis test followed by Dunn's test **(C)**, that did not show significant differences.

Remibrutinib and rilzabrutinib did not compromise Btk-independent pathways of platelet aggregation stimulated by GPCR activation with thrombin receptor-activating peptide (TRAP), arachidonic acid (AA), or ADP under the conditions tested (Figure 3).

### Effect of Remibrutinib and Rilzabrutinib on *in vitro* Bleeding Time

To investigate whether remibrutinib and rilzabrutinib might impair primary hemostasis, the platelet function analyzer PFA-200 was used. The instrument aspirates citrate-anticoagulated blood under constant vacuum from a reservoir through a capillary and a small hole in a membrane filter which was coated in our experiments with collagen and epinephrine (collagen/epinephrine cartridge). The time required to obtain full occlusion of the aperture is reported as “*in vitro* closure time” (32, 35). The PFA is used for routine screening of patients with potential hemorrhagic risk and is very sensitive to monitor aspirin intake (36, 37).

**Figure 4:**
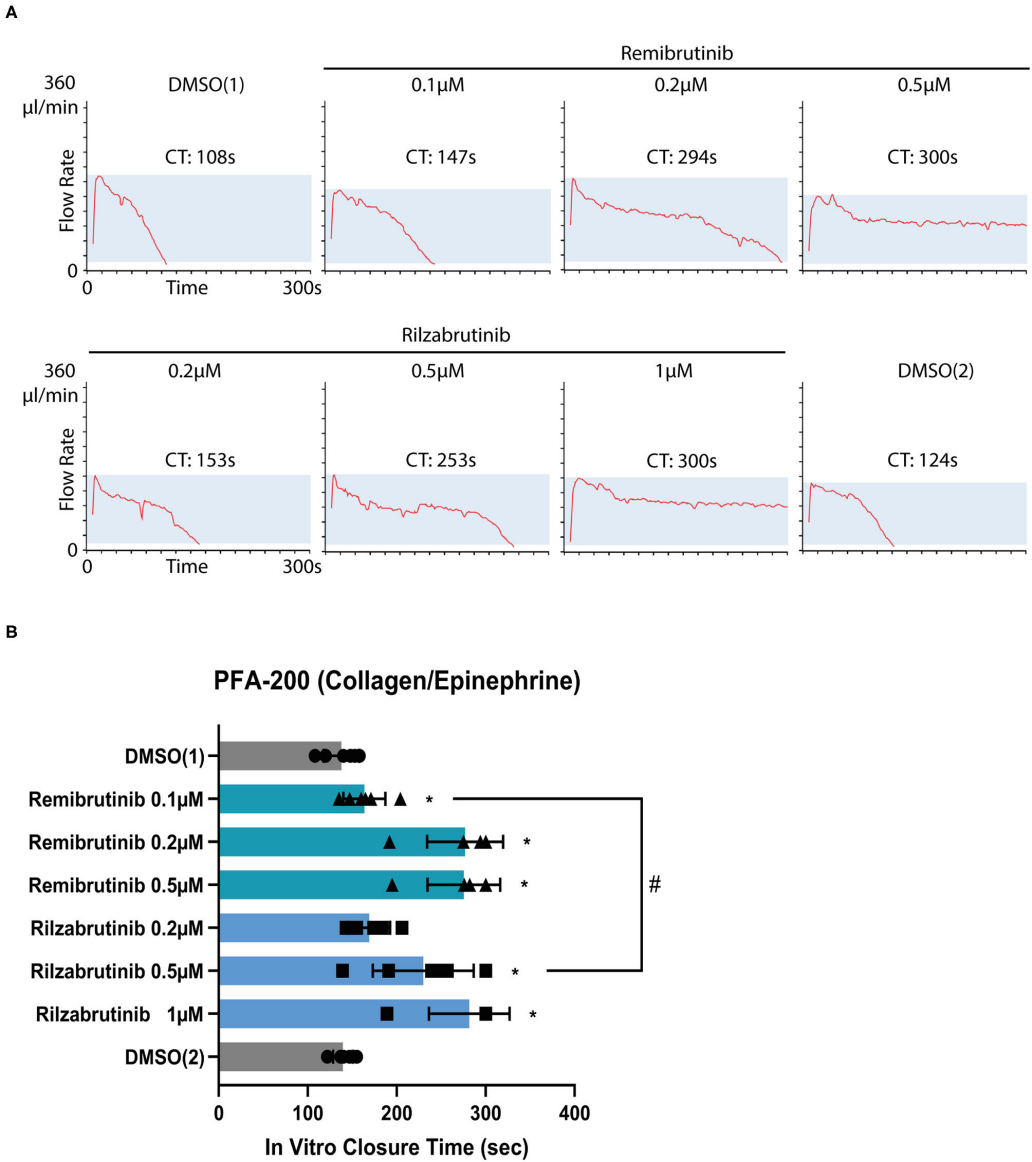
Effect of remibrutinib and rilzabrutinib on bleeding time *in vitro*. Citrate anticoagulated blood samples were pre-incubated for 1 h at 37°C with solvent (DMSO, 0.1%) or increasing concentrations of remibrutinib or rilzabrutinib and then transferred to collagen/epinephrine cartridges. The *in vitro* closure time (CT) was measured with the PFA-200. DMSO (1) and DMSO (2) control samples were measured at the beginning and end of the experiment, respectively. **(A)** Representative PFA-200 tracings. **(B)** The aligned dot plot bar charts show CT values of different concentrations of remibrutinib and rilzabrutinib. Values are mean ± SD (*n* = 6). Statistical analysis was carried out using the Wilcoxon matched-pairs signed rank test comparing against DMSO1 (^*^p < 0.05) or concentrations of remibrutinib (0.1 μM) and rilzabrutinib (0.5 μM) that inhibited Btk-dependent pathways of platelet aggregation by >90% (#*p* < 0.05).

Closure time was slightly, but significantly prolonged by 0.1 μM remibrutinib (Figures 4A,B) which suppressed >85% Btk-dependent platelet aggregation after GPVI activation with low dose collagen and after VWF/GPIb activation with ristocetin (Figures 1A, 2A,C), but it did not exceed the upper limit of the normal range (170 s). Higher concentrations of remibrutinib (0.2 and 0.5 μM) significantly and profoundly prolonged closure time.

For rilzabrutinib it was found that a concentration of 0.2 μM which inhibited GPVI-dependent plaque-stimulated platelet aggregation by 56% (Figure 1B) did not affect significantly the closure time. A concentration of 0.5 μM rilzabrutinib equipotent to 0.1 μM remibrutinib suppressed >90% Btk-dependent platelet aggregation after low dose collagen- and ristocetin-stimulated aggregation (Table 1; Figure 2) and significantly increased closure time by 67% (Figures 4A,B). The closure time was significanly more prolonged than by 0.1 μM remibrutinib (Figure 4B). A concentration of 1 μM rilzabrutinib prolonged bleeding time maximally. DMSO, the solvent of BTKi, did not affect closure time as shown previously (9), and the DMSO controls showed similar values at the beginning and the end of the experiments (Figures 4A,B).

**Table 1:** IC_50_ values of remibrutinib, rilzabrutinib and other BTK inhibitors for inhibition of low degree GPVI stimulated platelet aggregation, and comparison with therapeutic drug plasma levels.

**BTK Inhibitors**	**IC_50_ (μM)**	**Therapeutic drug** **plasma level (μM)**
Remibrutinib	0.03^*^	0.46^a^
Rilzabrutinib	0.16^*^	0.33^b^
Fenebrutinib	0.016^*^	0.6^c^
Ibrutinib	0.025^#^	0.31^d^
Zanubrutinib	0.094^#^	1.4^e^
Tirabrutinib	0.268^#^	1.96^f^
Acalabrutinib	0.372^#^	1.78^g^
Evobrutinib	1.20^#^	Not known

* *Hirudin-anticoagulated blood was pre-incubated with the BTKi for 1 h or 15 min (fenebrutinib, Supplementary Figure 1) prior to stimulation with plaque homogenate*.

# *Hirudin-anticoagulated blood was pre-incubated with the BTKi for 1 h before stimulation with low collagen concentrations (0.2–0.5 μg/ml). Platelet aggregation was measured by multiple electrode aggregometry (MEA)*.

a *Remibrutinib, 100 mg q.d, optimal dose in phase I study (38)*.

b *Rilzabrutinib, 300 mg b.i.d (39)*.

c *Fenebrutinib, 200 mg q.d (40)*.

d *Ibrutinib, 420 mg q.d (41)*.

e *Zanubrutinib, 320 mg q.d (42)*.

f *Tirabrutinib, 320 mg q.d (43)*.

g *Acalabrutinib, 100 mg b.i.d (44)*.

## Discussion

We demonstrate here in our study that (i) remibrutinib and rilzabrutinib inhibit and delay dose-dependently atherosclerotic plaque-induced GPVI-mediated platelet aggregation; (ii) remibrutinib (0.1 μM) and rilzabrutinib (0.5 μM) also block Btk-dependent GPVI-, GPIb/VWF- and FcγRIIa-stimulated platelet aggregation; (iii) higher concentrations of remibrutinib (≥0.2 μM) and therapeutic concentrations of rilzabrutinib (≥0.2 μM) prolong the bleeding time *in vitro* as measured by PFA-200.

According to the dose-response curve (Figures 1A,B), the potency for platelet inhibition of low degree GPVI-induced platelet aggregation of remibrutinib (IC_50_ = 0.03 μM) was 5 times higher than that of rilzabrutinib (IC_50_ = 0.16 μM). Compared with other BTKi (Table 1), remibrutinib is only slightly less potent than fenebrutinib (IC_50_ = 0.016 μM) and ibrutinib (IC_50_ = 0.025 μM) and more potent than zanubrutinib, rilzabrutinib, tirabrutinib, acalabrutinib and evobrutinib. The IC_50_ values of remibrutinib (IC_50_ = 0.03 μM) and rilzabrutinib (IC_50_ = 0.16 μM) are 12-times and 2-fold lower than the optimal plasma levels as determined in clinical phase 1 studies, respectively (38, 39). Additionally, both inhibitors induced a dose-dependent increase in delay of atherosclerotic plaque-induced aggregation that was associated with the suppression of aggregation in blood (Figure 1C). A delay was also shown in a previous study using ibrutinib- and acalabrutinib-treated washed platelets stimulated by collagen while the maximal aggregation was unaffected (17).

Remibrutinib (0.1 μM) and rilzabrutinib (0.5 μM) significantly suppressed by >90% GPVI-dependent aggregation on low dose collagen, GPIb/VWF-dependent aggregation on ristocetin stimulation, and FcγRIIA-dependent aggregation upon CD32 cross-linking, but it had no effect on TRAP, AA, or ADP stimulation as expected according to the results of previous studies with other BTKi (7, 9, 16, 17), although it has to be stated that a non-existing effect is difficult to prove and may depend on the experimental conditions.

High dose collagen GPVI-dependent aggregation was suppressed to a similar degree of about 30% with remibrutinib (0.1 μM), rilzabrutinib (0.5 μM) and the Btk-selective reversible BTKi fenebrutinib (0.1 μM) (Supplementary Figure 2). This may indicate that the concentrations and incubation conditions of remibrutinib and rilzabrutinib used are selective for inhibition of Btk and unlikely to also inhibit Tec in platelets. This is unexpected considering the potent inhibition of Tec by rilzabrutinib *in vitro* (22). Rilzabrutinib by inhibiting Tec in addition to Btk would have shut-off GPVI signaling after high collagen stimulation.

As shown in several studies, low-degree GPVI activation only depends on Btk, while high dose collagen-induced GPVI signaling is also dependent on Tec co-activation (9, 15, 19, 45). In a previous study, 50 nM fenebrutinib was applied and only suppression of platelet aggregation on low but not high dose collagen stimulation was observed (9). Our different results may be explained due to the higher concentration of fenebrutinib (0.1 μM) applied in our study providing a more complete inhibition of Btk.

Our results show that the remibrutinib concentration to fully inhibit Btk-dependent pathways of platelet aggregation (0.1 μM) is lower than the reported maximal plasma level (0.46 μM) in a phase I study after intake of 100 mg q.d. for 12 days (38). Thus, this concentration is expected to block completely Btk-dependent signaling in platelets *in vivo*. The equivalent rilzabrutinib concentration (0.5 μM) is higher than the plasma C_max_ reported in clinical studies after therapeutic dosage for autoimmune diseases (0.33 μM) (Table 1) (39). Since the IC_50_ of rilzabrutinib for inhibition of plaque-induced platelet aggregation in blood was lower (0.16 μM), it is likely that therapeutic concentrations of rilzabrutinib inhibit Btk-dependent pathways of platelet aggregation, but not entirely. Our results are in contrast to findings showing no inhibition of ristocetin- and high dose collagen- induced aggregation of platelet-rich plasma from healthy donors and ITP patients pre-incubated with 1 μM rilzabrutinib for 15 min *in vitro* (26, 46). The discrepancy might be explained by differences of the experimental system used (blood vs. PRP), different concentrations of collagen (low vs. high) and exposure times of rilzabrutinib (long vs. short). We selected a long exposure time (1 h), since this might better simulate the *in vivo* situation after absorption of the drug, and previous studies have shown that platelet inhibition with irreversible BTKi increases with the exposure time (17, 19).

Bleeding is a frequent side effect of treatment with certain irreversible BTKi such as ibrutinib and the second generation BTKi acalabrutinib, zanubrutinib, and tirabrutinib used to treat B-cell malignancies (5, 47–49). Exclusive inhibition of Btk should not increase bleeding since XLA patients who are deficient of Btk do not show an impairment of haemostasis (13). It has been discussed that bleeding by these BTKi is related to off-target inhibition of Tec, since this kinase is functionally involved in GPVI-induced platelet activation (8, 15). By comparison, for fenebrutinib, a reversible highly selective BTKi, which is the most selective BTKi and which shows no inhibition of Tec (50), no bleeding events were reported in clinical trials (non-Hodgkin lymphoma, chronic lymphocytic leukemia, rheumatoid arthritis, and systemic lupus erythematosus) (5). Also, fenebrutinib *in vitro*, even at very high concentrations up to 1 μM did not prolong bleeding time measured by the PFA-200 (9).

The reversible BTKi fenebrutinib binds to an inactive conformation of Btk (51). Also, remibrutinib which was developed from fenebrutinib-like scaffolds to bind to the inactive conformation of Btk (20, 51) showed a 175-fold higher affinity for Btk over Tec in binding assays *in vitro* (20). Thus, it was expected that remibrutinib would not increase *in vitro* bleeding time measured by PFA-200, similar to fenebrutinib (9). However, we observed that bleeding times *in vitro* were already slightly but significantly increased after blood incubation with 0.1 μM remibrutinib (which inhibited >90% of Btk-dependent pathways of platelet aggregation), and strongly prolonged by remibrutinib concentrations of 0.2 and 0.5 μM. The results for remibrutinib are similar to a previous study, in which low concentrations of the irreversible BTKi ibrutinib, zanubrutinib, acalabrutinib, and tirabrutinib inhibited GPVI- dependent platelet aggregation by >70%, but 2- to 2.5-fold higher concentrations of these BTKi were required to significantly increase the bleeding time *in vitro* (19). The increase of closure time was similar to that observed after treatment with low dose aspirin (52).

In a phase I placebo controlled clinical trial of remibrutinib (total 156 healthy subjects), mild self-limited bleeding events were observed only in 4 persons in the multiple-ascending dose cohorts with remibrutinib intake for 12 days. These included two subjects in the 600 mg q.d. cohort with epistaxis and two subjects in the 100 mg cohort with trauma-triggered hematomas (38).

Rilzabrutinib in our study slightly but non-significantly increased at 0.2 μM closure time in the PFA device, the increase was at 0.5 μM pronounced (Figure 4B). Potent Tec inhibition could contribute to the increased *in vitro* bleeding time (5); however, the results of the aggregation studies upon stimulation with high concentrations of collagen argue against simultaneous Tec inhibition in platelets by 0.5 μM of rilzabrutinib (see above).

Thus, the mechanisms underlying the increase of closure times elicited by remibrutinib as well as rilzabrutinib are unlikely to involve off-target inhibition of Tec. They could be related to effects on the Btk protein itself. Recently it was found that binding of certain irreversible BTKi (except fenebrutinib) to the kinase domain had long-range allosteric effects on the SH2-and SH3- regulatory domains changing their conformation toward an activated state of the protein (53).

In contrast to remibrutinib, there was not a clear difference of rilzabrutinib concentrations that inhibited Btk-dependent pathways of platelet aggregation in the MEA and robustly increased the closure time in the PFA; the concentration of rilzabrutinib of 0.5 μM does both. Maximal therapeutic concentrations of rilzabrutinib (0.33 μM) are expected to significantly increase the closure time in the PFA device, but no treatment-related bleeding had been noted in the ITP clinical trial with rilzabrutinib (25), although the median platelet count at study entry was only 14.173/μl (25). However, 7% (2/27) of patients treated with rilzabrutinib had treatment-related epistaxis as observed in the latest pemphigus clinical trial (24).

## Limitations

Although our *in vitro* study has the advantage of reducing the complexity of the experimental conditions, and the different effects of the two BTKi studied on platelets in blood are obvious, these data cannot directly be translated into the situation *in vivo*. Clinical studies of platelet function *ex vivo* after oral intake of therapeutic dosage are warranted to approach the *in vivo* effects of remibrutinib and rilzabrutinib on platelets.

## Conclusion

In the present study we found significant differences of the two BTKi remibrutinib and rilzabrutinib on platelets that would favor remibrutinib as a candidate for further development as an antiplatelet drug to inhibit Btk-dependent platelet activation pathways underlying atherothrombosis and certain platelet-related immune disorders. Since *de novo* protein synthesis in platelets is very limited and because low concentrations of irreversible BTKi such as remibrutinib may covalently inactivate platelet BTK already by a single exposure at low concentrations during absorption, it is likely that low doses of such a selective irreversible BTKi are effective in cardiovascular prevention without affecting the immune system (7, 8, 54). Our study further suggests that off-target effects on Tec are unlike to be involved in the increase of closure time measured by PFA, and may not explain the bleeding side effects elicited by BTKi.

## Data Availability Statement

The original contributions presented in the study are included in the article/Supplementary Material, further inquiries can be directed to the corresponding author/s.

## Ethics Statement

The studies involving human participants were reviewed and approved by Ethics Committee of the Faculty of Medicine of the University of Munich. Written informed consent for participation was not required for this study in accordance with the national legislation and the institutional requirements.

## Author Contributions

RD designed and performed experiments, analyzed data, and wrote the manuscript. LG performed experiments and analyzed data. RB provided plaque material. MS supervised experiments and contributed to discussions. CW supervised and contributed to discussions. WS conceived the study, designed experiments, and wrote the manuscript. PH supervised and analyzed experiments and wrote the manuscript. All authors have contributed significantly to this manuscript.

## Funding

This work was supported by the Deutsche Forschungsgemeinschaft, SFB1123, A2 (PH); RD was sponsored by the Ludwig-Maximilians-University (LMU)-China Scholarship Council (CSC) program.

## Conflict of Interest

The authors declare that the research was conducted in the absence of any commercial or financial relationships that could be construed as a potential conflict of interest.

## Publisher's Note

All claims expressed in this article are solely those of the authors and do not necessarily represent those of their affiliated organizations, or those of the publisher, the editors and the reviewers. Any product that may be evaluated in this article, or claim that may be made by its manufacturer, is not guaranteed or endorsed by the publisher.

## References

[1] Bruton OC. Agammaglobulinemia. Pediatrics. (1952) 9:722–8. 14929630

[2] Vetrie D Vorechovský I Sideras P Holland J Davies A Flinter F. The gene involved in X-linked agammaglobulinaemia is a member of the src family of protein-tyrosine kinases. Nature. (1993) 361:226–33. 10.1038/361226a0 8380905

[3] Tsukada S Saffran DC Rawlings DJ Parolini O Allen RC Klisak I. Deficient expression of a B cell cytoplasmic tyrosine kinase in human X-linked agammaglobulinemia. Cell. (1993) 72:279–90. 10.1016/0092-8674(93)90667-F 8425221

[4] Pal Singh S Dammeijer F Hendriks RW. Role of Bruton's tyrosine kinase in B cells and malignancies. Mol Cancer. (2018) 17:57. 10.1186/s12943-018-0779-z 29455639 PMC5817726

[5] von Hundelshausen P Siess W. Bleeding by Bruton Tyrosine kinase-inhibitors: dependency on drug type and disease. Cancers. (2021) 13:1103. 10.3390/cancers13051103 33806595 PMC7961939

[6] Futatani T Watanabe C Baba Y Tsukada S Ochs HD. Bruton's tyrosine kinase is present in normal platelets and its absence identifies patients with X-linked agammaglobulinaemia and carrier females. Br J Haematol. (2001) 114:141–9. 10.1046/j.1365-2141.2001.02905.x 11472359

[7] Busygina K Jamasbi J Seiler T Deckmyn H Weber C Brandl R. Oral Bruton tyrosine kinase inhibitors selectively block atherosclerotic plaque–triggered thrombus formation in humans. Blood. (2018) 131:2605–16. 10.1182/blood-2017-09-808808 29559479

[8] Busygina K Denzinger V Bernlochner I Weber C Lorenz R Siess W. Btk inhibitors as first oral atherothrombosis-selective antiplatelet drugs? Thromb Haemost. (2019) 119:1212–21. 10.1055/s-0039-1687877 31087308

[9] Goldmann L Duan R Kragh T Wittmann G Weber C Lorenz R. Oral Bruton tyrosine kinase inhibitors block activation of the platelet Fc receptor CD32a (FcgammaRIIA): a new option in HIT? Blood Adv. (2019) 3:4021–33. 10.1182/bloodadvances.2019000617 31809536 PMC6963242

[10] von Hundelshausen P Lorenz R Siess W Weber C. Vaccine-induced immune thrombotic thrombocytopenia (VITT): targeting pathomechanisms with Bruton Tyrosine kinase inhibitors. Thromb Haemost. (2021). 10.1055/a-1481-3039. [Epub ahead of print]. 33851389

[11] Payne H Ponomaryov T Watson SP Brill A. Mice with a deficiency in CLEC-2 are protected against deep vein thrombosis. Blood. (2017) 129:2013–20. 10.1182/blood-2016-09-742999 28104688 PMC5408561

[12] Nicolson PL Welsh JD Chauhan A Thomas MR Kahn ML Watson SP. A rationale for blocking thromboinflammation in COVID-19 with Btk inhibitors. Platelets. (2020) 31:685–90. 10.1080/09537104.2020.1775189 32552307

[13] Shillitoe B Gennery A. X-linked agammaglobulinaemia: outcomes in the modern era. Clin Immunol. (2017) 183:54–62. 10.1016/j.clim.2017.07.008 28729230

[14] Quek LS Bolen J Watson SP. A role for Bruton's tyrosine kinase (Btk) in platelet activation by collagen. Curr Biol. (1998) 8:1137–S1. 10.1016/S0960-9822(98)70471-3 9778529

[15] Atkinson BT Ellmeier W Watson SP. Tec regulates platelet activation by GPVI in the absence of Btk. Blood. (2003) 102:3592–9. 10.1182/blood-2003-04-1142 12842985

[16] Bye AP Unsworth AJ Desborough MJ Hildyard CAT Appleby N Bruce D. Severe platelet dysfunction in NHL patients receiving ibrutinib is absent in patients receiving acalabrutinib. Blood Adv. (2017) 1:2610–23. 10.1182/bloodadvances.2017011999 29296914 PMC5728643

[17] Nicolson PLR Hughes CE Watson S Nock SH Hardy AT Watson CN. Inhibition of Btk by Btk-specific concentrations of ibrutinib and acalabrutinib delays but does not block platelet aggregation mediated by glycoprotein VI. Haematologica. (2018) 103:2097–108. 10.3324/haematol.2018.193391 30026342 PMC6269309

[18] Jamasbi J Megens RT Bianchini M Uhland K Munch G Ungerer M. Cross-linking GPVI-Fc by anti-Fc antibodies potentiates its inhibition of atherosclerotic plaque- and collagen-induced platelet activation. JACC Basic Transl Sci. (2016) 1:131–42. 10.1016/j.jacbts.2016.03.008 27766315 PMC5063538

[19] Denzinger V Busygina K Jamasbi J Pekrul I Spannagl M Weber C. Optimizing platelet GPVI inhibition versus haemostatic impairment by the Btk inhibitors ibrutinib, acalabrutinib, ONO/GS-4059, BGB-3111 and evobrutinib. Thromb Haemost. (2019) 119:397–406. 10.1055/s-0039-1677744 30685871

[20] Angst D Gessier F Janser P Vulpetti A Walchli R Beerli C. Discovery of LOU064 (Remibrutinib), a potent and highly selective covalent inhibitor of Bruton's Tyrosine kinase. J Med Chem. (2020) 63:5102–18. 10.1021/acs.jmedchem.9b01916 32083858

[21] Hill R Smith P Krishnarajah J Bradshaw J Masjedizadeh M Bisconte A. Discovery of PRN1008, a novel, reversible covalent btk inhibitor in clinical development for rheumatoid arthritis: abstract number: 1671. Arthr Rheumatol. (2015) 67:2062–3.

[22] Murrell D Gourlay S Hill R Bisconte A Francesco M Smith P. Development of PRN1008, a novel, reversible covalent BTK inhibitor in clinical development for pemphigus. in Proceedings of the Medical Dermatology Society Annual Meeting, Washington, DC, USA. (2016). p. 3.

[23] Bradshaw JM McFarland JM Paavilainen VO Bisconte A Tam D Phan VT. Prolonged and tunable residence time using reversible covalent kinase inhibitors. Nat Chem Biol. (2015) 11:525–31. 10.1038/nchembio.1817 26006010 PMC4472506

[24] Murrell DF Patsatsi A Stavropoulos P Baum S Zeeli T Kern JS. Proof of concept for the clinical effects of oral rilzabrutinib, the first Bruton tyrosine kinase inhibitor for pemphigus vulgaris: the phase II BELIEVE study. Br J Dermatol. (2021). 10.1111/bjd.20431. [Epub ahead of print]. 33942286 PMC8518737

[25] Kuter D Boccia R Lee E-J Efraim M Tzvetkov N Mayer J. Phase I/II, open-label, adaptive study of oral Bruton Tyrosine kinase inhibitor PRN1008 in patients with relapsed/refractory primary or secondary immune thrombocytopenia. Blood. (2019) 134:87–7. 10.1182/blood-2019-122336

[26] Langrish CL Bradshaw JM Owens TD Campbell RL Francesco MR Karr DE. PRN1008, a reversible covalent BTK inhibitor in clinical development for immune thrombocytopenic purpura. Blood. (2017) 130:1052–2. 10.1182/blood.V130.Suppl_1.1052.105228705838

[27] Reininger AJ Bernlochner I Penz SM Ravanat C Smethurst P Farndale RW. A 2-step mechanism of arterial thrombus formation induced by human atherosclerotic plaques. J Am Coll Cardiol. (2010) 55:1147–58. 10.1016/j.jacc.2009.11.051 20223370

[28] Penz S Reininger AJ Brandl R Goyal P Rabie T Bernlochner I. Human atheromatous plaques stimulate thrombus formation by activating platelet glycoprotein VI. FASEB J. (2005) 19:898–909. 10.1096/fj.04-2748com 15923400

[29] Tóth O Calatzis A Penz S Losonczy H Siess W. Multiple electrode aggregometry: a new device to measure platelet aggregation in whole blood. Thromb Haemost. (2017) 96:781–8. 10.1160/TH06-05-0242 17139373

[30] von Pape K-W Aland E Bohner J. Platelet function analysis with PFA-100® in patients medicated with acetylsalicylic acid strongly depends on concentration of sodium citrate used for anticoagulation of blood sample??Presented in part at the 43rd annual meeting of the GTH, February 25, 1999, Mannheim, Germany. Thromb Res. (2000) 98:295–9. 10.1016/S0049-3848(99)00236-4 10822076

[31] Bampalis VG Brantl SA Siess W. Why and how to eliminate spontaneous platelet aggregation in blood measured by multiple electrode aggregometry. J Thromb Haemost. (2012) 10:1710–4. 10.1111/j.1538-7836.2012.04819.x 22712617

[32] Kundu SK Heilmann EJ Sio R Garcia C Davidson RM Ostgaard RA. Description of an in vitro platelet function analyzer–PFA-100. Semin Thromb Hemost. (1995) 21 (Suppl. 2):106–12. 10.1055/s-0032-1313612 7660150

[33] Favaloro EJ. Clinical utility of the PFA-100. Semin Thromb Hemost. (2008) 34:709–33. 10.1055/s-0029-1145254 19214910

[34] Schulz C Penz S Hoffmann C Langer H Gillitzer A Schneider S. Platelet GPVI binds to collagenous structures in the core region of human atheromatous plaque and is critical for atheroprogression in vivo. Basic Res Cardiol. (2008) 103:356–67. 10.1007/s00395-008-0722-3 18431526

[35] Kratzer MAA Negrescu EV Hirai A Yeo YK Petra F Siess W. The thrombostat system a useful method to test antiplatelet drugs and diets. Semin Thromb Hemost. (1995) 21:25–31. 10.1055/s-0032-1313599 7660154

[36] Marshall PW Williams AJ Dixon RM Growcott JW Warburton S Armstrong J. A comparison of the effects of aspirin on bleeding time measured using the Simplate method and closure time measured using the PFA-100, in healthy volunteers. Br J Clin Pharmacol. (1997) 44:151–5. 10.1046/j.1365-2125.1997.00639.x 9278200 PMC2042813

[37] Ng KF Lawmin JC Tsang SF Tang WM Chiu KY. Value of a single preoperative PFA-100 measurement in assessing the risk of bleeding in patients taking cyclooxygenase inhibitors and undergoing total knee replacement. Br J Anaesth. (2009) 102:779–84. 10.1093/bja/aep091 19411670

[38] Kaul M End P Cabanski M Schuhler C Jakab A Kistowska M. Remibrutinib (LOU064): a selective potent oral BTK inhibitor with promising clinical safety and pharmacodynamics in a randomized phase I trial. Clin Transl Sci. (2021). 10.1111/cts.13005. [Epub ahead of print]. 33834628 PMC8504815

[39] Smith PF Krishnarajah J Nunn PA Hill RJ Karr D Tam D. A phase I trial of PRN1008, a novel reversible covalent inhibitor of Bruton's tyrosine kinase, in healthy volunteers. Br J Clin Pharmacol. (2017) 83:2367–76. 10.1111/bcp.13351 28636208 PMC5651318

[40] Byrd JC Smith S Wagner-Johnston N Sharman J Chen AI Advani R. First-in-human phase 1 study of the BTK inhibitor GDC-0853 in relapsed or refractory B-cell NHL and CLL. Oncotarget. (2018) 9:13023–13035. 10.18632/oncotarget.24310 29560128 PMC5849192

[41] Chen J Kinoshita T Gururaja T Sukbuntherng J James D Lu D. The effect of Bruton's tyrosine kinase (BTK) inhibitors on collagen-induced platelet aggregation, BTK, and tyrosine kinase expressed in hepatocellular carcinoma (TEC). Eur J Haematol. (2018) 101:604–12. 10.1111/ejh.13148 30030853

[42] Tam CS Trotman J Opat S Burger JA Cull G Gottlieb D. Phase 1 study of the selective BTK inhibitor zanubrutinib in B-cell malignancies and safety and efficacy evaluation in CLL. Blood. (2019) 134:851–9. 10.1182/blood.2019001160 31340982 PMC6742923

[43] Walter HS Rule SA Dyer MJ Karlin L Jones C Cazin B. A phase 1 clinical trial of the selective BTK inhibitor ONO/GS-4059 in relapsed and refractory mature B-cell malignancies. Blood. (2016) 127:411–9. 10.1182/blood-2015-08-664086 26542378 PMC4731845

[44] Byrd JC Harrington B O'Brien S Jones JA Schuh A Devereux S. Acalabrutinib (ACP-196) in relapsed chronic lymphocytic leukemia. N Engl J Med. (2016) 374:323–32. 10.1056/NEJMoa1509981 26641137 PMC4862586

[45] Oda A Ikeda Y Ochs HD Druker BJ Ozaki K Handa M. Rapid tyrosine phosphorylation and activation of Bruton's tyrosine/Tec kinases in platelets induced by collagen binding or CD32 cross-linking. Blood. (2000) 95:1663–70. 10.1182/blood.V95.5.1663.005k44_1663_1670 10688822

[46] Langrish CL Bradshaw JM Francesco MR Owens TD Xing Y Shu J. Preclinical efficacy and anti-inflammatory mechanisms of action of the Bruton Tyrosine kinase inhibitor rilzabrutinib for immune-mediated disease. J Immunol. (2021) 206:1454–68. 10.4049/jimmunol.2001130 33674445 PMC7980532

[47] Shatzel JJ Olson SR Tao DL McCarty OJT Danilov AV DeLoughery TG. Ibrutinib-associated bleeding: pathogenesis, management and risk reduction strategies. J Thromb Haemost. (2017) 15:835–47. 10.1111/jth.13651 28182323 PMC6152914

[48] Sibaud V Beylot-Barry M Protin C Vigarios E Recher C Ysebaert L. Dermatological toxicities of Bruton's Tyrosine kinase inhibitors. Am J Clin Dermatol. (2020) 21:799–812. 10.1007/s40257-020-00535-x 32613545

[49] Sekiguchi N Rai S Munakata W Suzuki K Handa H Shibayama H. A multicenter, open-label, phase II study of tirabrutinib (ONO/GS-4059) in patients with Waldenstrom's macroglobulinemia. Cancer Sci. (2020) 111:3327–37. 10.1111/cas.14561 32639651 PMC7469793

[50] Crawford JJ Johnson AR Misner DL Belmont LD Castanedo G Choy R. Discovery of GDC-0853: a potent, selective, and noncovalent bruton's tyrosine kinase inhibitor in early clinical development. J Med Chem. (2018) 61:2227–45. 10.1021/acs.jmedchem.7b01712 29457982

[51] Gabizon R London N. A fast and clean BTK inhibitor. J Med Chem. (2020) 63:5100–1. 10.1021/acs.jmedchem.0c00597 32401033 PMC7304894

[52] Reny JL De Moerloose P Dauzat M Fontana P. Use of the PFA-100 closure time to predict cardiovascular events in aspirin-treated cardiovascular patients: a systematic review and meta-analysis. J Thromb Haemost. (2008) 6:444–50. 10.1111/j.1538-7836.2008.02897.x 18194417

[53] Joseph RE Amatya N Fulton DB Engen JA-O Wales TE Andreotti AA-O. Differential impact of BTK active site inhibitors on the conformational state of full-length BTK. eLife. 9:e60470. 10.7554/eLife.60470 33226337 PMC7834017

[54] Payrastre B Ribes A. Low-dose Btk inhibitors: an ‘aspirin' of tomorrow? Haematologica. (2021) 106:2–4. 10.3324/haematol.2020.265173 33386711 PMC7776254

