# Supplementary material for: Effects of the Btk-Inhibitors Remibrutinib (LOU064) and Rilzabrutinib (PRN1008) With Varying Btk Selectivity Over Tec on Platelet Aggregation and in vitro Bleeding Time
Source: Front Cardiovasc Med. 2021 Sep 24;8:749022. doi: 10.3389/fcvm.2021.749022 (PMC8498029; doi:10.3389/fcvm.2021.749022)
Supplement: Supplementary file 1 [file Data_Sheet_1.docx]

**Supplemental Figure 1**


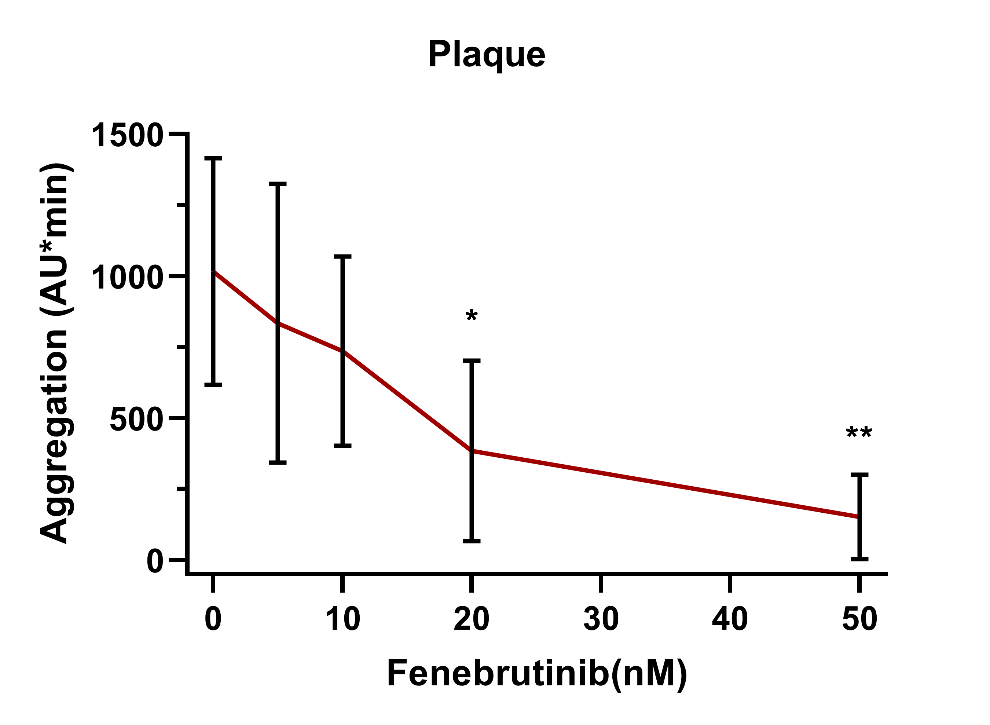


**Supplemental Figure 1. Dose-response curve of fenebrutinib on plaque homogenate- induced platelet aggregation.** Hirudin anticoagulated blood were preincubated with solvent (DMSO, 0.1%) or increasing concentrations of fenebrutinib for 15min at 37°C before stimulation with plaque homogenate (833µg/ml). Statistical analysis was carried out comparing against baseline (without BTKi) using the ordinary one-way ANOVA followed by Bonferroni’s test. Values are mean ± SD (n=5). **p*˂0.05, ***p*˂0.01.

**Supplemental Figure 2**


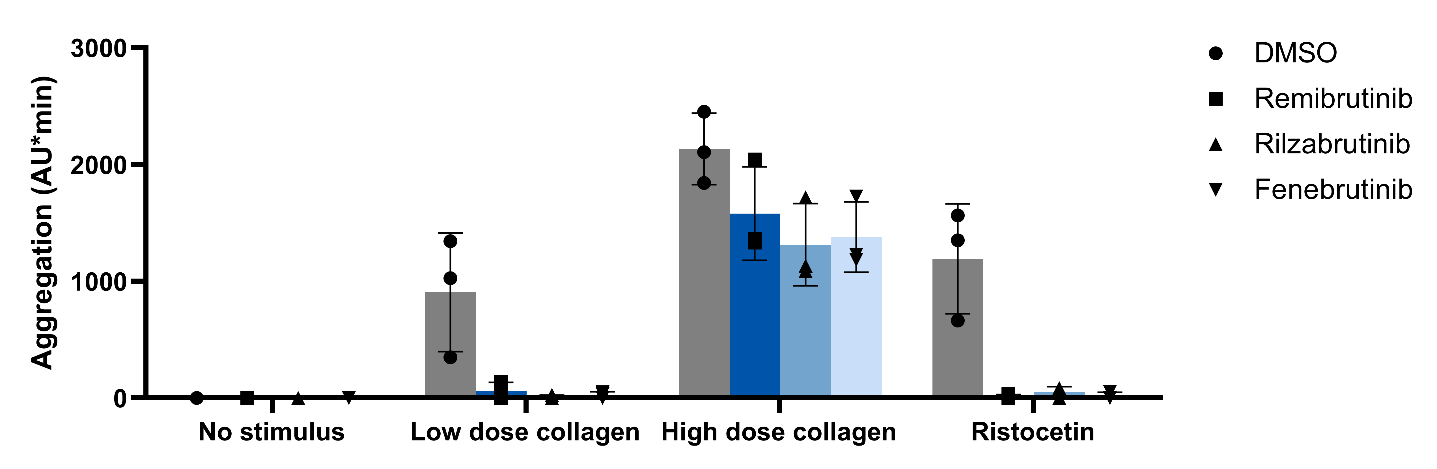


**Supplemental Figure 2. Comparison of remibrutinib and rilzabrutinib with fenebrutinib on** **inhibition of GPVI- and GPIb/VWF- dependent platelet aggregation.** Hirudin anticoagulated blood samples were treated with solvent control (DMSO, 0.1%), 0.1 µM remibrutinib, 0.5µM rilzabrutinib or 0.1µM fenebrutinib for 1 hour at 37°C prior to stimulation for 10 min with low dose collagen (0.4-0.6µg/ml), high dose collagen (4-6µg/ml), or ristocetin (0.5mg/ml). Bar graphs show the effect of BTKi on platelet aggregation upon stimulation (n=3).
